# Advances in Detecting Low Prevalence Somatic *TERT* Promoter Mutations in Papillary Thyroid Carcinoma

**DOI:** 10.3389/fendo.2021.643151

**Published:** 2021-03-12

**Authors:** Vitor Rodrigues da Costa, Larissa Valdemarin Bim, Luiza Dornelles Penteado Pacheco e Silva, Gabriel Avelar Colloza-Gama, André Uchimura Bastos, Rosana Delcelo, Gisele Oler, Janete Maria Cerutti

**Affiliations:** ^1^ Genetic Bases of Thyroid Tumors Laboratory, Department of Morphology and Genetics, Division of Genetics, Universidade Federal de São Paulo, São Paulo, Brazil; ^2^ Repare DNA Laboratory, Biomedical Sciences Institute, Universidade de São Paulo, São Paulo, Brazil; ^3^ Department of Pathology, Universidade Federal de São Paulo, São Paulo, Brazil

**Keywords:** Papillary thyroid carcinoma, TERT (telomerase reverse transcriptase), C250T, C228T, droplet digital PCR, TaqMan allele discrimination assay

## Abstract

**Background:**

Two recurrent *TERT* (telomerase reverse transcriptase) promoter mutations, C228T and C250T, have been reported in thyroid carcinomas and were correlated with high-risk clinicopathological features and a worse prognosis. Although far more frequent in the poorly differentiated and undifferentiated thyroid cancer, the *TERT* promoter mutations play a significant role on PTC recurrence and disease-specific mortality. However, the prevalence varies considerably through studies and it is uncertain if these differences are due to population variation or the methodology used to detect *TERT* mutations. In this study we aim to compare three different strategies to detect *TERT* promoter mutations in PTC.

**Methods:**

DNA was isolated from formalin-fixed paraffin-embedded (FFPE) specimens from 89 PTC and 40 paired lymph node metastases. The prevalence of the hot spot *TERT* C228T and C250T mutations was assessed in FFPE samples using TaqMan SNP genotyping assays. Random samples were tested by Sanger Sequencing and droplet digital PCR (ddPCR).

**Results:**

In general, 16 out of 89 (18%) PTC samples and 14 out of 40 (35%) lymph node metastases harbored *TERT* promoter mutations by TaqMan assay. Sanger sequencing, performed in random selected samples, failed to detect *TERT* mutations in four samples that were positive by TaqMan SNP genotyping assay. Remarkably, ddPCR assay allowed detection of *TERT* promoter mutations in six samples that harbor very low mutant allele frequency (≤ 2%) and were negative by both genotype assay and Sanger Sequencing.

**Conclusion:**

This study observed a good concordance among the methodologies used to detect *TERT* promoter mutations when a high percentage of mutated alleles was present. Sanger analysis demonstrated a limit of detection for mutated alleles. Therefore, the prevalence of *TERT* promoter mutations in PTC may be higher than previously reported, since most studies have conventionally used Sanger sequencing. The efficient characterization of genetic alterations that are used as preoperative or postoperative diagnostic, risk stratification of the patient and individualized treatment decisions, mainly in highly heterogeneous tumors, require highly sensitive and specific approaches.

## Introduction

Telomeres are structures located at the end of chromosomes composed of DNA tandem repeat 5’-(TTAGGG)n-’3 sequences associated with a protein sheltering complex. These structures act as a capping system to protect the chromosome, avoiding end-to-end chromosome fusions and its recognition as a site of DNA damage, promoting the maintenance of genome stability (1, 2). In normal healthy cells, with the absence of telomere maintenance mechanisms, the telomeres progressively shorten within each division process.

In normal somatic adult cells, the critical shortening of telomere Length (TL) triggers DNA damage response and induces replicative arrest, cellular senescence or apoptosis (2–4). Although cancer cells can bypass senescence-associated cell cycle arrest beyond their normal lifespan, they continue to replicate until entering in a crisis state, a period with high rates of apoptosis due to gross chromosomal abnormalities and genome instability (1, 2, 4). The scape from this crisis state requires the activation of Telomerase Reverse Transcriptase (hTERT) giving these cells unlimited replicative potential. Therefore, telomere length and telomerase activity are crucial for cancer progression (2, 5, 6). The main mechanism for the maintenance of TL is the expression of the hTERT, encoded by *TERT* gene (2).

The hTERT expression is up-regulated *via* genetic mechanisms including *TERT* amplifications, hTERT structural variants, *TERT* promoter mutations and epigenetic mechanism such as *TERT* promoter hipomethylation (7, 8). Recently, two C>T mutations in the promoter of *TERT* gene, located at -124 and -146 bp upstream of *TERT*’s translational start site and designated as C228T and C250T, have been described in several cancer types, including thyroid carcinomas (9, 10). These somatic mutations in *TERT* promoter create *de novo* consensus binding sites (CCGGAA) for the E-twenty-six (ETS) transcription factor family, providing a mechanism for cancer-specific activation of hTERT expression (3). Recent studies have investigated the role of *TERT* promoter mutations in cancer diagnosis, prognosis, and predictor of radiotherapy resistance (6, 11).

In thyroid cancer, *TERT* promoter mutations have been proposed as prognostic markers, associated with decreased disease-free survival (6, 9, 12–14). Additionally, several studies performed in European (15), Asian (16) and in North American populations (10) have suggested that the co-occurrence of *TERT* promoter mutations and *BRAF* V600E or RAS mutations contributes to the progression of thyroid cancer and, therefore, have great value in indicating a worse outcome (12, 13, 17–22). The mechanism of this synergistic effect can be explained, at least in part, by the fact that MAPK-induced ETS factors selectively bind to the mutant *TERT* promoter

Although studies from different countries and geographical regions around the world have consistently found *TERT* promoter mutations in thyroid cancers and provided evidence of their role in thyroid cancer progression, the prevalence of *TERT* mutations varies considerably. It is not clear whether the differences in the prevalence observed are due to geographical variation or the method used to detect *TERT* mutations (21, 23–25). As an example, in PTC cohort, the prevalence of C228T mutation ranges from 7–23% (mean 9.7%) and the C250T mutation ranges from 0–10% (mean 2.1%) (9, 25).

Given that the presence of *TERT* promoter mutations have important clinical implications on diagnosis, prognosis and treatment of thyroid cancer and that different methods are known to have distinctive sensitivities, in the present study we investigated the prevalence of *TERT* promoter mutation in a Cohort of PTC using three different strategies.

## Materials and Methods

### Tumor Samples

The study was performed on available archival formalin-fixed, paraffin-embedded (FFPE) blocks selected from primary PTC (*n* = 89) and paired lymph node metastases (*n* = 40) from patients who underwent surgical resection at the Hospital São Paulo, Universidade Federal de São Paulo (UNIFESP). In six PTC cases, two paired lymph node metastases were evaluated (**Table 1**). All FFPE tissue sections, obtained from the Department of Pathology, UNIFESP, were reviewed by an onsite pathologist (RD) to confirm the diagnosis. For cases with multifocality, the largest tumor focus was used. Essentially, all PTC samples and lymph node metastases showed over 70% of tumor cellularity.

**Table 1:** TERT status in primary PTC and its paired lymph node metastasis.

ID	Primary PTC	ID	Lymph node metastasis
**1**	C228T	**1**	WT
**6**	WT	**2**	C228T
**9**	WT	**3**	WT
**10**	C228T	**4**	C228T
**11**	WT	**5**	WT
**15**	WT	**6**	C228T
**16**	WT	**7**	WT
**30**	WT	**8**	WT
**32**	C228T	**9**	WT
**33**	WT	**10**	WT
35*	C250T	**11**	C250T
35*	C250T	**12**	C250T
38*	C228T	**13**	WT
38*	C228T	**14**	WT
**39**	WT	**15**	C250T
40*	WT	**16**	C228T
40*	WT	**17**	C250T
**48**	C228T	**18**	WT
**51**	WT	**19**	WT
**52**	WT	**20**	C250T
53*	WT	**21**	WT
53*	WT	**22**	WT
**54**	WT	**23**	WT
**55**	C228T	**24**	C250T
57*	WT	**25**	WT
57*	WT	**26**	WT
**68**	WT	**27**	WT
**73**	WT	**28**	C250T
**74**	WT	**29**	WT
**76**	WT	**30**	WT
78*	WT	**31**	WT
78*	WT	**32**	WT
**79**	C228T	**33**	WT
**80**	WT	**34**	WT
**81**	WT	**35**	WT
**82**	WT	**36**	C228T
**83**	WT	**37**	WT
**84**	WT	**38**	WT
**85**	C228T	**39**	C228T

*Six PTC cases whose 2 lymph node metastases were evaluated.

### Cell Lines

To test whether TaqMan SNP genotyping assay is able to properly distinguish wild-type allele from mutated alleles, we initially tested human papillary (TPC1) and undifferentiated (8505C) thyroid carcinoma cells that are positive for different TERT promoter mutations and a follicular thyroid carcinoma cell (WRO) that is negative for both *TERT* promoter mutations as controls (10). The thyroid carcinoma cell lines were maintained at 37°C in a 5% CO_2_ humidified atmosphere, as described (26). Short tandem repeat (STR) profiling was performed for the cell line authentication and to check for cross-contamination.

### DNA Isolation From FFPE Samples and Cell Lines

Tissues dissected from three 10-μm-thick slices of paraffin-embedded tissue specimens were treated with xylene washes at 65°C to remove paraffin with subsequent absolute and 70% ethanol washes to remove xylene. DNA from primary tumor and metastases samples was extracted using the NucleoSpin Tissue Kit (Macherey-Nagel, Duren, Germany), according to the manufacturer’s instructions and quantified in a NanoDrop Spectrophotometer (ThermoFisher Scientific, Waltham, MA, USA). DNA from cell lines was isolated as previously described (27).

### Screening of *TERT* Promoter Mutations in PTC Using Allelic Discrimination Assay

To identify the two most prevalent *TERT* promoter mutations (C250T or C228T), we used two TaqMan SNP genotyping assays, which reliably discriminate the mutant from the wild-type alleles (Cat#A44177). The assay Hs000000092_rm detects the TERT C228T (-124bp upstream of the ATG start codon) and includes a mutation-specific TaqMan probe (FAM fluorescence AGGGGAGGGGCTGGGAGGGCCCGGA**[A]**GGGGCTGGGCCGGGGACCCGGGAGG). The assay Hs000000093_rm detects the TERT C250T (-146bp upstream of the ATG start codon) and includes a mutation-specific TaqMan probe (FAM fluorescence GGAGGGGGCTGGGCCGGGGACCCGG**[A]**AGGGGTCGGGACGGGGCGGGGTCCG). Each assay includes also a wild-type TaqMan probe directed to the same region (VIC fluorescent AGGGGAGGGGCTGGGAGGGCCCGGA**[G]**GGGGCTGGGCCGGGGACCCGGGAGG) and PCR primer pairs to amplify the sequence of interest. The PCR reaction mixture for each assay consisted of 30 ng of DNA, 1X TaqMan Genotyping Master Mix and 1X Custom TaqMan *TERT* mutation assay (C228T or C250T) in a final reaction volume of 20 μl. The PCR reaction was performed on QuantStudio 12K Flex Real-Time PCR System (Applied Biosystems, Thermo Fisher Scientific). The cycling condition was 95°C for 10 min, 55 cycles of 95°C for 15 s and 60°C for 1 min. For each sample, the qPCR reaction was performed in duplicate. Allelic discrimination analysis was performed using the 12K Flex software.

### *TERT* Promoter Mutations in PTC by Sanger Sequencing

To endorse the TaqMan SNP genotyping assay results, 46 samples were selected to undergo nested PCR followed by Sanger sequencing. For the first-round, the amplification of the promoter region of the *TERT* gene containing both the C250T and C228T mutations was carried out using the kit OneTaq Polymerase (New England BioLabs, Ipswich, MA, USA). The PCR reaction was performed using primers designed as follows: sense 5’-AGTGGATTCGCGGGCACAGA-3’ and antisense 5’-CAGCGCTGCCTGAAACTC-3’, yielding a 181 bp fragment. The reaction was performed using 50–100 ng of DNA, under the following steps: 95°C for 3 min, 10 cycles of a three-step program of 95°C for 30 s, 57°C for 30 s and 68°C for 1 min, 25 cycles of a three-step program of 95°C for 40 s, 57°C for 40 s, 68°C for 70 s and final amplification of 68°C for 7 min. Second-round was performed using 1 μl of the product of the first reaction under the following steps: 95°C for 3 min, 35 cycles of a three-step program of 95°C for 30 s, 57°C for 30 s, 68°C for 1 min, and final amplification of 68°C for 7 min. The primers were designed as follows: sense 5’-GTCCTGCCCCTTCACCTTC-3’ and antisense 5’-CAGCGCTGCCTGAAACTC-3’, yielding a 165 bp product. The PCR products from the second-round were analyzed by gel electrophoresis on a 2.0% agarose gel, visualized in the Gel Doc EZ system (Bio-Rad, Hercules, CA, USA), and purified using illustra ExoProStar S (GE Healthcare, Waukesha, WI, USA) prior to sequencing. The second-round purified product was sequenced using the Big Dye TerminatorCycle v3.1 Sequencing Ready Reaction kit in the ABI 3130 sequencer, according to manufacturer’s instructions (Applied Biosystems, Foster City, CA, USA). The electropherograms were analyzed using BioEdit (HALL, 1999) and CLC Sequence Analyzer (CLC bio, Aarhus, Denmark) softwares. All samples were sequenced twice.

### Quantification of *TERT* Promoter Mutations by Droplet Digital PCR (ddPCR)

The ddPCR analysis was performed using the QX100Droplet Digital PCR System (Bio-Rad) and the same custom TaqMan primers and probes for detection of *TERT* C250T and C228T promoter mutation (ThermoFisher Scientific) described in the allelic discrimination assay section. A known positive and negative DNA was included as control in each ddPCR run. The PCR reaction was performed using 1X ddPCR Super Mix, 1X TaqMan primer/probe for each *TERT* mutation, restriction enzyme *Hind*III (3U) and 30 ng of DNA to a final reaction volume of 20 μL. The PCR mixture was loaded into the QX100Droplet Digital PCR chip. The cycling condition was 96°C for 10 min, followed by 55 cycles of 98°C for 30 s and 55°C for 2 min.

The analysis and quantification of wild type and mutated alleles were performed using the QuantaSoft software, version 1.7.4.0917, and two ddPCR package software, version 1.12.0 (28). The threshold for analysis eligibility was the minimum of 10,000 detected droplets. The selection of positive samples was determined first by manual classification by subtracting all negative control detected droplets from samples of their respective plates on QuantaSoft software grid classification. After that, we applied the algorithm of R based package two ddPCR on all samples (28). For that, three positive samples with minimum noise have been selected and used as parameters to classify droplets in all samples under k-Nearest Neighbors (k-NN) algorithm classification. Also, we applied the rain classification parameter, where hard to classify droplets are excluded from analyses by a defined distance.

## Results

### *TERT* Promoter Mutation Specificity

*TERT* TaqMan SNP genotyping assay validation on qPCR was assessed by analyzing DNA samples from three thyroid carcinoma cell lines to ensure there was no cross-reactivity and because these cell lines had approximately 50% of mutant allele frequency. As expected, the papillary thyroid carcinoma cell line (TPC1) harbored the C228T mutation in heterozygosis. The undifferentiated thyroid carcinoma cell line (8505C) presented the C250T mutation in heterozygosis and the follicular thyroid carcinoma cell line (WRO) was wild-type for both *TERT* promoter mutations.

### Prevalence of C228T and C250T *TERT* Promoter Mutations Using Allelic Discrimination Assay

Overall, 16 out of 89 (18%) PTC samples were positive for *TERT* promoter mutations. The C228T mutation was found in 13 (14%) PTC samples, while 3 samples (3.4%) harbored the C250T mutation. The two mutations were mutually exclusive. When we compared the metastatic PTC (*n*=33), which had paired lymph node metastases available, we observed that 8/33 (24%) of primary tumors were positive for *TERT* promoter mutations, while 14/40 (35%) of paired lymph node metastases harbored *TERT* promoter mutations. Hence, the lymph node metastases showed a higher rate of *TERT* mutation (**Table 1** **;** **Supplementary Table 1**).

### *TERT* Mutations by Sanger Sequencing May Lead to False Negative Results

**Figure 1:**
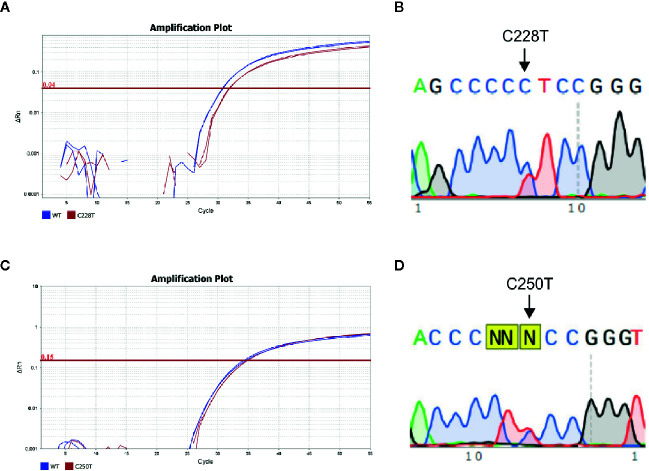
*TERT* promoter mutations in PTC specimens. **(A)** Amplification plot and **(B)** Electropherogram of a PTC case positive for the C228T *TERT* mutation using allele specific TaqMan assay and Sanger Sequencing, respectively; **(C)** amplification plot and **(D)** electropherogram of a PTC case positive for the C250T *TERT* mutation, using allelic specific TaqMan assay and Sanger Sequencing, respectively. The amplification curve of Wild type and mutated allele are represented in blue and red, respectively. The arrows indicate the point C>T mutations at C228 and C250 position.

As a high percentage of the DNA isolated from FFPE did not amplify by PCR, 46 samples (33 PTC and 13 lymph node metastases) were used to compare the sensitivity of TaqMan SNP genotyping assays *versus* Sanger Sequencing (**Figure 1**). There was an agreement between the results of allelic discrimination assay and Sanger Sequencing for most cases (**Supplementary Table 1**). However, Sanger sequencing failed to detect *TERT* promoter mutations in four (8%) samples that were positive by TaqMan SNP genotyping assay. By contrast, only one C228T positive case by Sanger Sequencing was negative by TaqMan SNP genotyping assay. Interestingly, this sample showed an additional mutation downstream to the C228T mutation site, which likely affected the annealing of the TaqMan probe for C228T mutation.

### ddPCR Shows Better Performance for Detecting *TERT* Mutations

**Figure 2:**
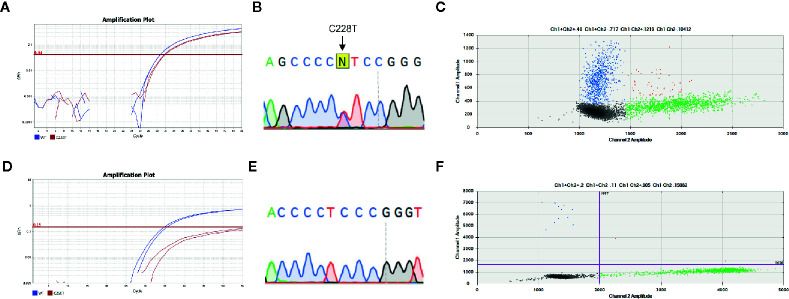
*TERT* promoter mutations detected by all assays used. Representative analysis of concordant mutations detected by **(A)** allelic specific TaqMan assay, **(B)** Sanger Sequencing and **(C)** droplet digital (dd)PCR. All assays detected *TERT* C228T mutation. A high abundance (41%) of fluorescent probe that detected *TERT* C228 mutation (blue) and wild-type probe (green) were observed, as exemplified by the count of droplets within each specific probe. A representative case with discordant result is shown **(D)** Allelic specific TaqMan assay and **(E)** Sanger Sequencing results where *TERT* C250T promoter mutation was not detected, while it was detected by **(F)** ddPCR. A low rate of mutated alleles (1.5%) is observed. The amplification curves in allelic specific TaqMan assay of wild type and mutated allele are represented in blue and red, respectively. The arrows indicate the regions expected to have the point mutations. The 2D amplitude plots of ddPCR analysis show the fluorescence associated with wild type probes (green), mutated (blue) or both alleles (orange).

We next tested 20 primary PTC and 10 paired lymph node metastases that were negative for *TERT* mutations to assess *TERT* mutational status using the droplet digital PCR (ddPCR) assay. The analysis of samples that were found to be wild type for these mutations by TaqMan SNP genotyping assay and Sanger Sequencing unveiled six cases (2/20 primary PTC and 4/10 lymph node metastases) that were positive for *TERT* mutation by ddPCR. Therefore, ddPCR detected *TERT* promoter mutation in additional 6/30 samples (20%) (**Figure 2**, **Tables 1** and **2**).

**Table 2:** *TERT* mutation status across different strategies.

	Allele Discrimination (Taqman Analysis)	Sanger Sequencing
False negative	6	8
False Positive	0	0
Concordant samples	5	3
Total positive in the ddPCR	11	11

Although we did not test all samples by ddPCR, it showed better performance in detecting *TERT* promoter mutation. Importantly, ddPCR used the same custom TaqMan primers and probes used for qPCR allelic discrimination assay. In view of the percentage of false negative results and considering that ddPCR is a digital technology that enables an absolute measure of target DNA with high precision, we took into consideration the percentage of the mutated alleles. The *TERT* mutant allele frequencies in the six false negative samples measured by ddPCR were 0.5, 2, 0.92, 1.5, 0.68, and 0.96% (**Supplementary Table 1**).

**Figure 3:**
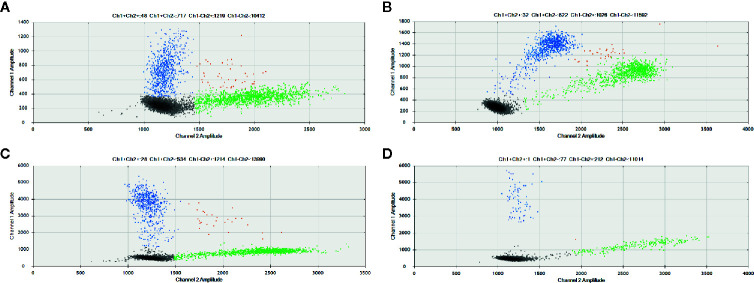
*TERT* promoter mutation detected by ddPCR in Primary PTC and paired lymph node metastasis. Results showed that mutant allele abundance was lower in the **(A)** primary PTC (41%) than in the **(B)** paired lymph node metastasis (44%). In other cases, the mutant allele abundance was higher in the **(C)** primary tumor than in the **(D)** paired metastasis (33 vs. 24%). The 2D amplitude plots of ddPCR analysis show the fluorescence associated with wild-type probes (green), mutated (blue) or both alleles (orange).

As control, we additionally selected five samples (two PTC and three lymph node metastases) that were positive for *TERT* mutations according to TaqMan SNP genotyping assays. Remarkably, the three cases that tested positive by both Sanger Sequencing and TaqMan SNP genotyping assay were positive for *TERT* mutation by ddPCR at allele frequencies of 33, 41, and 44% (**Figure 3**). Two cases that were positive only by Taqman SNP genotyping assays, had allele frequencies of 24 and 26% by ddPCR (**Supplementary Table 1** **)**.

Although some primary tumors have discordant results with their respective metastasis, most of them are multifocal PTC (9/13; 70%) (**Table 1** **;** **Supplementary Table 1**), and only the largest tumor focus was analyzed for each primary tumor. This explains, at least in part, the discordant results observed between primary tumor and paired lymph node metastasis.

## Discussion

Detecting mutations in the promoter region of *TERT* has been a puzzling task because of the high GC content of this region (nearly 80%). Therefore, specific and efficient PCR amplification of this target region is challenging. Moreover, the amplified region comprises two mutations that are separated by 22 bp and the sequences adjacent each mutation are almost identical to each other, which facilitate the formation of secondary structures and prevents DNA polymerase to copy these regions. In fact, due to this challenging task, our study has several limitations. We were not able to evaluate all samples using Sanger Sequencing.

As *TERT* promoter mutations are rule-in markers of thyroid malignant features and key prognostic markers, and as the occurrence of a mutation is a robust predictor of recurrences and worse outcome, a methodology that can reliably detect* *low frequency mutation*s* in routine preoperative or even postoperative diagnosis becomes increasingly important.

Up to now, most studies used Sanger sequencing for detecting *TERT* promoter mutations (10, 15, 29), which requires a mutant allele frequency of at least 20% to generate a positive signal. In this study we focused on detection of *TERT* mutation using TaqMan allelic specific amplification. We next compared the results to that obtained from Sanger Sequencing and ddPCR analysis. Both TaqMan allelic specific amplification by qPCR and ddPCR technique detected mutations that were missed by Sanger sequencing. One likely reason for the differences in PCR-based sensitivity may be amplicon length and the mutant allele fraction (MAF).

We found that there is a good concordance among the three methodologies when the samples show a high percentage (>30%) of mutated alleles. TaqMan allelic specific genotyping assays presented an improved performance when compared to Sanger sequencing, as it detected *TERT* mutations in cases with <30% of mutated alleles (**Supplementary Table 1**). Nonetheless, one limitation of the TaqMan assay is that it did not provide additional information about DNA sequence. While Sanger examines the entire sequence, is not quantitative and shows lower sensitivity. As far as we know, one study in medulloblastoma reported a TaqMan-based genotyping assay to detect recurrent *TERT* promoter mutations (30).

Analogous to TaqMan assays, the ddPCR assay is limited to individual mutation. However, digital methods have been shown to improve specificity and sensitivity of mutation detection through partition of the template DNA into 10,000–20,000 individual PCR reactions in one single sample (31).

In this study, we showed that ddPCR is highly sensitive and provides absolute quantification by direct counting positive wells, therefore may be a better choice to detect the presence of low-abundance recurrent mutations using either the invasive or noninvasive methods for the diagnosis or prognosis of cancer. The discrepancy between allelic discrimination assay and ddPCR results is likely related to the detection of lower allelic fraction mutations by ddPCR. The divergence between the primary tumor and their respective lymph node metastases is presumably linked to tumor heterogeneity. It has been shown that *TERT* promoter mutations confer an aggressive feature to the tumor, and it is likely that even cells with very low allelic fraction mutation for *TERT* will have replicative advantage. Therefore, early detection of *TERT* mutation may be of great importance for diagnosis, prognosis, and treatment decisions.

Similar to our findings, few studies have used ddPCR to identify the prevalence of TERT promoter mutation in blood and urinary cell-free tumor DNA and FFPE specimens to monitor melanoma (32–34), urothelial cancer (35), glioma (36), and thyroid (37). In thyroid, ddPCR was used to investigated *TERT* promoter mutations in follicular thyroid tumors of uncertain malignant potential (38). Several cases showed spatial heterogeneity, in which different regions of the primary tumor displayed either the C228T or the C250T mutation. ddPCR showed that C228T and C250T might co-occur in the same regions of the tumor. Ultimately, the authors detected sub-clonal mutations occurring in <10% of the tumor that Sanger sequencing reported as wild type (37). Yet, a much lower limit of detection (<0.1) was previously reported (25, 38, 39). The same is true for other emerging technologies that also show higher sensitivity than Sanger Sequencing, such as next generation sequencing (NGS), that have been used to identify genetic alteration in genes other than *TERT* and that have been associated with aggressive thyroid tumors subtype (40). Nevertheless, there is lack of data in the literature regarding the relevance of low percentages of mutations detected using either NGS or ddPCR in key genes associated with PTC progression and, therefore, their prognostic value.

Of note, ddPCR is a promising platform to absolute quantification of copy number alteration, a distinct mechanism that results in *TERT* upregulation and increased activity and that has been documented in thyroid carcinomas (13, 41).

Altogether, we believe that the prevalence of *TERT* promoter mutation in PTC is likely higher than previously reported, since specimens with a percentage of mutated alleles lower than 20% are probably not detected by Sanger sequencing assay. This study emphasizes the use of ddPCR into clinical practice, mainly in the context of aggressive and advanced thyroid carcinomas. Importantly, more studies are necessary to evaluate the percentage of mutated *TERT* promoter alleles and its correlation with the disease status in patients.

## Data Availability Statement

The raw data supporting the conclusions of this article will be made available by the authors, without undue reservation.

## Ethics Statement 

The studies involving human participants were reviewed and approved by Review Boards and Research Ethical Committees of UNIFESP (1309/11). Written informed consent for participation was not required for this study in accordance with the national legislation and the institutional requirements.

## Author Contributions

Conceptualization, VC, LB, GO, and JC. Data acquisition, VC, LB, LP, RD, and AB. Data analysis and interpretation, VC, LB, LP, AB, GO, and GAC-G. Original draft preparation, VC and LB. Review and editing, AB, GO, and JC. Supervision and funding acquisition, JC. All authors contributed to the article and approved the submitted version.

## Funding

The study was supported by research grants from The São Paulo State Research Foundation (FAPESP, 2014/06570-6). VC (2016\25127-1), GAC-G (2018/13203-0), AUB (2012/06221-6), and GO (2012/17545-7) were recipients of fellowship grants from FAPESP. JC is a recipient of a scholarship of Research Productivity from CNPq (304534/2018-8). We acknowledge the Coordenação de Aperfeiçoamento de Pessoal de Nível Superior (CAPES), for their financial support.

## Conflict of Interest

The authors declare that the research was conducted in the absence of any commercial or financial relationships that could be construed as a potential conflict of interest.
