# Supplementary material for: Advances in Detecting Low Prevalence Somatic TERT Promoter Mutations in Papillary Thyroid Carcinoma
Source: Front Endocrinol (Lausanne). 2021 Mar 12;12:643151. doi: 10.3389/fendo.2021.643151 (PMC7994758; doi:10.3389/fendo.2021.643151)
Supplement: Supplementary file 1 [file Table_1.docx]

**Supplementary Table 1:** *TERT* mutation status across different strategies

| **Sample ID** | **Sample** | **Multifocality** |  | **C228T** |  |  | **C250T** |  | **TERT mutation** |
| --- | --- | --- | --- | --- | --- | --- | --- | --- | --- |
|  |  |  | **Sanger** | **qPCR** | **ddPCR (%)** | **Sanger** | **qPCR** | **ddPCR( %)** |  |
| 1 | PTC | Y | - | C228T | - | - | WT | WT | C228T |
| 2 | PTC | N | - | WT | WT | - | WT | - | WT |
| 3 | PTC | NA | WT | WT | WT | WT | WT | - | WT |
| 4 | PTC | N | WT | WT | - | WT | WT | - | WT |
| 5 | PTC | Y | WT | WT | C228T (0.5) | WT | WT | - | C228T |
| 6 | PTC | Y | - | WT | - | - | WT | - | WT |
| 7 | PTC | Y | WT | WT | WT | WT | WT | - | WT |
| 8 | PTC | Y | - | WT | - | - | WT | - | WT |
| 9 | PTC | Y | - | WT | - | - | WT | - | WT |
| 10 | PTC | Y | C228T | C228T | C228T (41) | WT | WT | - | C228T |
| 11 | PTC | Y | - | WT | WT | - | WT | - | WT |
| 12 | PTC | N | WT | WT | WT | WT | WT | - | WT |
| 13 | PTC | Y | - | WT | - | - | WT | - | WT |
| 14 | PTC | Y | WT | WT | - | WT | WT | - | WT |
| 15 | PTC | Y | WT | WT | - | WT | WT | - | WT |
| 16 | PTC | Y | WT | WT | - | WT | WT | - | WT |
| 17 | PTC | N | - | WT | - | - | WT | - | WT |
| 18 | PTC | Y | - | WT | - | - | WT | - | WT |
| 19 | PTC | Y | WT | WT | - | WT | WT | - | WT |
| 20 | PTC | NA | WT | WT | - | WT | WT | - | WT |
| 21 | PTC | Y | WT | WT | - | WT | WT | - | WT |
| 22 | PTC | N | WT | WT | - | WT | WT | - | WT |
| 23 | PTC | N | - | WT | - | - | WT | - | WT |
| 24 | PTC | Y | WT | WT | - | WT | WT | - | WT |
| 25 | PTC | Y | - | WT | - | - | WT | - | WT |
| 26 | PTC | Y | WT | WT | - | WT | WT | - | WT |
| 27 | PTC | Y | WT | WT | - | WT | WT | - | WT |
| 28 | PTC | N | - | WT | - | - | WT | - | WT |
| 29 | PTC | Y | WT | WT | - | WT | WT | - | WT |
| 30 | PTC | Y | WT | WT | - | WT | WT | - | WT |
| 31 | PTC | Y | WT | WT | - | WT | WT | - | WT |
| 32 | PTC | N | - | C228T | - | - | WT | - | C228T |
| 33 | PTC | Y | - | WT | - | - | WT | - | WT |
| 34 | PTC | Y | WT | WT | - | WT | WT | - | WT |
| 35 | PTC | Y | WT | WT | - | C250T | C250T | C250T (32.8) | C250T |
| 36 | PTC | Y | WT | WT | - | WT | WT | - | WT |
| 37 | PTC | Y | - | WT | - | - | WT | - | WT |
| 38 | PTC | N | WT | C228T | - | WT | WT | - | C228T |
| 39 | PTC | N | WT | WT | - | WT | WT | - | WT |
| 40 | PTC | Y | WT | WT | - | WT | WT | - | WT |
| 41* | PTC | NA | C228T | WT | - | WT |  | - | C228T |
| 42 | PTC | NA | - | C228T | - | - | WT | - | C228T |
| 43 | PTC | N | C228T | C228T | - | WT | WT | - | C228T |
| 44 | PTC | NA | - | WT | - | - | WT | - | WT |
| 45 | PTC | N | - | C228T | - | - | WT | - | C228T |
| 46 | PTC | NA | WT | WT | - | WT | WT | - | WT |
| 47 | PTC | Y | - | WT | - | - | WT | - | WT |
| 48 | PTC | Y | - | C228T | - | - | WT | - | C228T |
| 49 | PTC | NA | - | WT | - | - | WT | - | WT |
| 50 | PTC | Y | - | WT | - | - | WT | - | WT |
| 51 | PTC | N | - | WT | - | - | WT | - | WT |
| 52 | PTC | Y | - | WT | - | - | WT | - | WT |
| 53 | PTC | N | - | WT | - | - | WT | - | WT |
| 54 | PTC | N | - | WT | - | - | WT | - | WT |
| 55 | PTC | Y | C228T | C228T | - | WT | WT | - | C228T |
| 56 | PTC | Y | - | WT | - | - | WT | - | WT |
| 57 | PTC | Y | WT | WT | - | WT | WT | - | WT |
| 58 | PTC | Y | - | WT | - | - | WT | - | WT |
| 59 | PTC | N | - | WT | - | - | WT | - | WT |
| 60 | PTC | Y | WT | C228T | - | WT | WT | - | C228T |
| 61 | PTC | N | - | WT | - | - | WT | - | WT |
| 62 | PTC | Y | - | WT | - | - | WT | - | WT |
| 63 | PTC | N | - | WT | - | - | WT | - | WT |
| 64 | PTC | N | - | WT | - | - | WT | - | WT |
| 65 | PTC | Y | - | WT | - | - | WT | - | WT |
| 66 | PTC | N | - | WT | - | - | WT | - | WT |
| 67 | PTC | N | WT | WT | - | WT | WT | - | WT |
| 68 | PTC | N | - | WT | - | - | WT | WT | WT |
| 69 | PTC | NA | - | WT | - | - | WT | - | WT |
| 70 | PTC | N | - | WT | - | - | WT | WT | WT |
| 71 | PTC | NA | - | WT | - | - | WT | C250T (2.0) | C250T |
| 72 | PTC | NA | - | WT | - | - | WT | - | WT |
| 73 | PTC | N | - | WT | - | - | WT | WT | WT |
| 74 | PTC | Y | - | WT | - | - | WT | WT | WT |
| 75 | PTC | N | WT | WT | - | WT | WT | WT | WT |
| 76 | PTC | N | - | WT | - | - | WT | - | WT |
| 77 | PTC | N | - | WT | - | - | WT | - | WT |
| 78 | PTC | Y | - | WT | - | - | WT | WT | WT |
| 79 | PTC | Y | - | C228T | - | - | WT | WT | C228T |
| 80 | PTC | Y | - | WT | - | - | WT | WT | WT |
| 81 | PTC | Y | - | WT | - | - | WT | WT | WT |
| 82 | PTC | Y | - | WT | - | - | WT | WT | WT |
| 83 | PTC | Y | - | WT | - | - | WT | WT | WT |
| 84 | PTC | Y | - | WT | - | - | WT | WT | WT |
| 85 | PTC | N | - | C228T | - | - | WT | WT | C228T |
| 86 | PTC | Y | - | WT | - | - | WT | WT | WT |
| 87 | PTC | N | - | WT | - | - | WT | - | WT |
| 88 | PTC | Y | - | WT | - | - | WT | WT | WT |
| 89 | PTC | Y | - | WT | - | - | WT | WT | WT |
| 1 | Metastasis | - | - | WT | - | - | WT | WT | WT |
| 2 | Metastasis | - | C228T | C228T | - | WT | WT | - | C228T |
| 3 | Metastasis | - | WT | C228T | - | WT | WT | - | WT |
| 4 | Metastasis | - | C228T | C228T | C228T (44.5) | WT | WT | - | C228T |
| 5 | Metastasis | - | WT | WT | - | WT | WT | - | WT |
| 6 | Metastasis | - | WT | WT | - | WT | WT | - | C228T |
| 7 | Metastasis | - | - | WT | - | - | WT | - | WT |
| 8 | Metastasis | - | - | WT | - | - | WT | - | WT |
| 9 | Metastasis | - | WT | WT | - | WT | WT | - | WT |
| 10 | Metastasis | - | WT | C228T | - | WT | WT | - | WT |
| 11 | Metastasis | - | WT | WT | - | WT | WT | C250T (24.4) | C250T |
| 12 | Metastasis | - | - | WT | - | - | C250T | C250T (26.6) | C250T |
| 13 | Metastasis | - | - | WT | - | - | WT | - | WT |
| 14 | Metastasis | - | - | WT | - | - | WT | - | WT |
| 15 | Metastasis | - | - | WT | - | - | WT | C250T (1.5) | C250T |
| 16 | Metastasis | - | WT | C228T | - | WT | WT | - | C228T |
| 17 | Metastasis | - | - | WT | - | - | WT | C250T (0.68) | C250T |
| 18 | Metastasis | - | - | WT | - | - | WT | - | WT |
| 19 | Metastasis | - | - | WT | - | - | WT | - | WT |
| 20 | Metastasis | - | - | WT | - | - | WT | - | C250T |
| 21 | Metastasis | - | WT | WT | - | WT | WT | - | WT |
| 22 | Metastasis | - | - | C228T | - | - | WT | - | WT |
| 23 | Metastasis | - | - | WT | - | - | WT |  | WT |
| 24 | Metastasis | - | WT | WT | - | WT | WT | C250T (0.96) | C250T |
| 25 | Metastasis | - | WT | WT | - | WT | WT |  | WT |
| 26 | Metastasis | - | - | WT | - | - | WT |  | WT |
| 27 | Metastasis | - | - | WT | - | - | WT |  | WT |
| 28 | Metastasis | - | - | WT | - | - | WT | C250T (0.91) | C250T |
| 29 | Metastasis | - | - | WT | - | - | WT | WT | WT |
| 30 | Metastasis | - | - | WT | - | - | WT | - | WT |
| 31 | Metastasis | - | - | WT | - | - | WT | - | WT |
| 32 | Metastasis | - | - | WT | - | - | WT | WT | WT |
| 33 | Metastasis | - | - | WT | - | - | WT | WT | WT |
| 34 | Metastasis | - | - | WT | - | - | WT | - | WT |
| 35 | Metastasis | - | - | WT | - | - | WT | WT | WT |
| 36 | Metastasis | - | WT | C228T | - | WT | WT | WT | C228T |
| 37 | Metastasis | - | - | WT | - | - | WT | WT | WT |
| 38 | Metastasis | - | - | WT | - | - | WT | WT | WT |
| 39 | Metastasis | - | - | C228T | - | - | WT | - | C228T |

WT: wild type. (-): Not determined. NA: Not Available. * Sample that showed additional mutation by Sanger
